# Extracellular Vesicles in Viral Infections of the Nervous System

**DOI:** 10.3390/v12070700

**Published:** 2020-06-28

**Authors:** Naseer A. Kutchy, Eric S. Peeples, Susmita Sil, Ke Liao, Ernest T. Chivero, Guoku Hu, Shilpa Buch

**Affiliations:** 1 Department of Pharmacology and Experimental Neuroscience, University of Nebraska Medical Center, Omaha, NE 68198-5880, USA; naseer.kutchy@unmc.edu (N.A.K.); susmita.sil@unmc.edu (S.S.); ke.liao@unmc.edu (K.L.); ernest.chivero@unmc.edu (E.T.C.); guoku.hu@unmc.edu (G.H.); 2 Department of Pediatrics, University of Nebraska Medical Center, Omaha, NE 68198, USA; epeeples@childrensomaha.org

**Keywords:** virus, HIV-1, Zika, exosome, neurodegenerative diseases

## Abstract

Almost all types of cells release extracellular vesicles (EVs) into the extracellular space. EVs such as exosomes and microvesicles are membrane-bound vesicles ranging in size from 30 to 1000 nm in diameter. Under normal conditions, EVs mediate cell to cell as well as inter-organ communication via the shuttling of their cargoes which include RNA, DNA and proteins. Under pathological conditions, however, the number, size and content of EVs are found to be altered and have been shown to play crucial roles in disease progression. Emerging studies have demonstrated that EVs are involved in many aspects of viral infection-mediated neurodegenerative diseases. In the current review, we will describe the interactions between EV biogenesis and the release of virus particles while also reviewing the role of EVs in various viral infections, such as HIV-1, HTLV, Zika, CMV, EBV, Hepatitis B and C, JCV, and HSV-1. We will also discuss the potential uses of EVs and their cargoes as biomarkers and therapeutic vehicles for viral infections.

## 1. Introduction

Living cells release a wide variety of extracellular vesicles (EVs) containing specific cargoes of proteins, RNA and DNA into the extracellular space. Following their release, EVs can then either be taken up by the neighboring cells or can travel to distant recipient cells via the body fluids. EVs have been found to be secreted in large numbers in most body fluids, including blood, breast milk, sweat, saliva, ascites fluid, urine and cerebrospinal fluid (CSF) [1,2,3,4,5].

Alterations in the numbers and size of EVs as well as their cargo content has been implicated in various infectious neurodegenerative diseases [6]. The EV content released from normal cells versus those from infected cells varies significantly in its composition [7]. These differences in their distinctive characteristics could be effectively tapped for diagnostic purposes. Various studies have reported that viruses manipulate EV biogenesis to their advantage, thus aiding the viruses to be more infective and thereby also facilitating disease transmission and pathogenesis [7,8]. It must also be noted that upon infection by the virus, the host immune cells also produce EVs which play an important role in mounting of the viral immune response. For example, EVs released from B lymphocytes, have been shown to contain class II major histocompatibility (MHC-II)-antigen complexes which, in turn, promotes the EVs to activate CD4+ T cells in an antigen-specific manner [9]. In addition, studies have demonstrated that EVs released by dendritic cells contain class I major histocompatibility (MHC-I)-peptide complex that stimulates cytotoxic CD8+ T cells. EVs can thus influence both cell-dependent and independent mechanisms for presenting antigens to given cells and play a significant role in regulating the cells involved in the generation of the adaptive immune response [10,11,12]. 

EVs derived from viral infected cells have shown to significantly impact the immune system via various mechanisms, including but not limited to, generation of the adaptive immune responses. Investigating the pathways affected by the virus-induced EVs and uncovering their mechanism of action could thus be of value in the development of therapeutic agents aimed at targeting the pathogens. In addition, EVs carrying pathogen-derived factors are of significant interest for use as diagnostic biomarkers in a given viral infection [13]. As opposed to the identification of free proteins and RNA in body fluids, packaging in EVs protects the proteins and RNA from circulating hydrolase activity [14], potentially resulting in improved diagnostic sensitivity. Furthermore, EVs are stable in circulating body fluids, can shuttle a wide variety of biomolecules and drugs, and are very selective biogenic carriers, making them ideally suited as carriers for therapeutic interventions [15,16,17,18,19,20,21].

## 2. Extracellular Vesicles

Eukaryotic cells are capable of secreting membrane-bound EVs of different sizes carrying heterogeneous cargo contents within them. EVs as a group are highly heterogeneous and research suggests that there could be multiple other EV subtypes. The field of EV research field has gained significant momentum from researchers all over the world owing to their ability to act as cell signaling mediators, and has also garnered increased interest in the fields of cell biology, biotechnology and pharmaceutical industry.

### 2.1. Biogenesis of EVs

**Figure 1:**
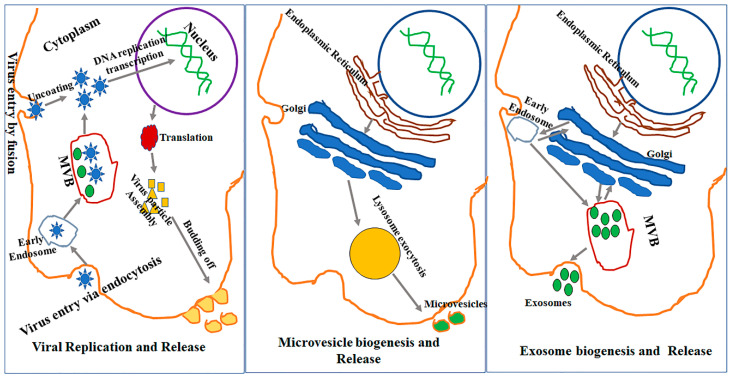
Virus replication and release share intracellular pathways with microvesicle and exosome biogenesis and release. Most of the cellular machinery involving the genome, endoplasmic reticulum, Golgi complex, lysosomes, late endosome, and multivesicular bodies (MVBs) are mutually used by viruses to replicate inside the cell and by the host cell for the generation of extracellular vesicles (EVs) such as microvesicles and exosomes.

The exosome biogenesis pathway involves inward budding of the late endosomal membrane, developing into intraluminal vesicles (ILVs) (Figure 1). There are multiple pathways leading to the production of ILVs, some of which are dependent on the Endosomal Sorting Complex Required for Transport (ESCRT) while others are ESCRT-independent. The ESCRT is an important system in exosome biogenesis involving several ESCRT proteins such as Tsg101 and Alix, which are also widely used as signature exosomal markers [22,23]. The ESCRT is composed of four proteins complexes (ESCRT-0, I, II, III), Alix, and other proteins with defined function in exosome biogenesis. All of the ESCRT related proteins are involved sequentially in the exosome biogenesis process. ESCRT-0 is involved in the recognition of cargo, ESCRT-0, I and II in cargo recruitment and membrane invagination, ESCRT-III in vesicle maturation and neck constriction, and ATPase Vps4 in inward membrane scission [24]. The ESCRT-III proteins can form filaments, flat spirals, tubes and conical funnels, which are thought to direct membrane remodeling and scission. Additionally, while the ESCRT-III protein assembly and disassembly are dependent on ATPase VPS4, mechanism(s) of scission have not yet been fully understood. Recently, Friand et al. [25] reported that an alternative ESCRT-dependent pathway relies heavenly on Alix (an ESCRT accessory protein) that interacts with both syndecan (a heparin sulphate) and syntenin (a member of the PDZ protein). Syndecan is internalized from the cell surface of endosomes and interacts with cytosolic surface protein syntenin which, in turn, binds to Alix and other ESCRT proteins to promote inward membrane invagination [25]. 

In addition, studies in which ESCRT components where inactivated led to the discovery of additional EV biogenesis pathways other than ESCRT [26]. One ESCRT-independent mechanism has been shown to involve the lipid neutral sphingomyelinase (nSMase) which is an essential component for the production of ceramide on the surface of the endosomal membrane. nSMase has a conic shape which promotes the generation of ILVs by forming negative curvature [27]. Another ESCRT-independent mechanism for EV biogenesis involves tetraspanins, which specifically promote the sorting of specific proteins into EVs and their clustering on the surfaces of late endosomes (MVBs) [27]. For example, the PreMelanosome protein (PMEL) is sorted into ILVs by a process involving CD63 tetraspanin [28,29,30]. 

According to a report by Simons and Raposo [1], multiple mechanisms are involved in the secretion of EVs and the inhibition of one single pathway does not completely block the EV release, thus supporting independent roles in EV biogenesis. For the biogenesis of exosomes, ESCRT-independent and ESCRT-dependent pathways could, at times, act synergistically, as in the case of phospholipid scramblase 3 which is secreted in exosomes by synergism between ESCRT and the ceramide pathway [31].

A completely different mechanism of EV biogenesis results in the release of microvesicles. It is believed that microvesicles are generated from direct shedding of the plasma membrane and are released promptly after their production [32]. The cargo to be carried out by the microvesicles is accumulated on the cytosolic face of the plasma membrane, after which the outward membrane curvature starts. The small GTPase Arf6 and GTPases of Rho family proteins then aid in creating the scission by contracting actin localized under the plasma membrane [33]. The microvesicle formation creates specific and localized changes in the plasma membrane lipids and its protein composition.

### 2.2. EV Biogenesis versus Virus Budding

There are several similarities between the biogenesis of EVs and the release of virus particles from host cells. For instance, both processes involve the ESCRT complex and its associated proteins, suggesting the possibility of an interaction between the virus life cycle within a cell and EV biogenesis/release pathways [7]. Briefly, the typical process of virus budding starts with the virus having its capsid (envelope protein) attach to its receptor on the cell membrane. Once the virus attaches to the cellular membrane, it is endocytosed by a mechanism that is analogous to the formation of early endosomes. Certain enveloped viruses, however, do not go through endocytosis and instead directly enter inside the cell after their fusion with the cell membrane. Once the viral nucleic acid of some RNA viruses is inside the cell, it incorporates into the host cell genome, allowing it to hijack and use the host machinery for its replication and/or transcription. This exploitation of host machinery amplifies virus particles inside the cell. As these particles accumulate, they either leave the infected cell by budding from the cell membrane—in a manner similar to microvesicle release—or they kill the host cell and are subsequently released following loss of the cell membrane integrity. 

Virus budding has been reported as a primary mechanism for the release of virus particles outside the cell for some viruses, including human immunodeficiency virus (HIV) and an exocytic pathway in the hepatitis C virus (HCV) [34]. The HIV virus recruits ESCRT-1 protein components and Alix using its structural protein, Gag, for virus particle assembly and release [35]. The mechanism of action of Herpesviruses is to exploit the ESCRT pathway proteins by acquiring the trans-Golgi network (TGN) and endosomes prior to their release into the extracellular space [34,36]. There is conclusive evidence that virus-infected cells release infective virions, EVs, and other non-replicative particles that contain viral elements or defective virus particles and exhibit similar characteristics to EVs. It is currently challenging, however, to identify which specific pathway is operating under what circumstance inside the cell and how the cells decide which pathway should be prioritized under a given condition. This is particularly complex when a cell has undergone viral infection owing to the similarities between EV biogenesis and viral budding (Figure 1) [37].

### 2.3. Viral Infection and EV Release

Viruses and EVs share an important evolutionary role, which is to deliver their genetic material from one cell to another—a mechanism that allows viruses to integrate into the cellular genome which, in turn, can be stably inheritable (Figure 1) [38,39,40,41]. Similar to the enveloped viruses, EVs can also shuttle their genetic material, proteins and other molecules into recipient cells, leading to altered transcription and functional change in the latter cells. Another similarity between the EVs and viruses lies in the composition of the virus envelope proteins and EV surface proteins which determines their respective adhesion to the plasma membrane of the given target cells. For example, EV-enriched proteins—tetraspanins—along with lipids, facilitate specific targeting of vesicles to recipient cells and also determine their ability to fuse with the plasma membranes [42]. Furthermore, common cellular proteins, including the tetraspanins, are shared by the EVs and viruses have been shown to be critical of the ability of the virus to infect target cells and determine their infectivity [43]. It is also suggested that, owing to the similarity between EVs and viruses, many viruses have developed the ability to hijack host cell EVs to increase viral infectivity. As an example, EVs can be used by the viruses to transport the machinery such as reverse transcriptase, DNA polymerases and transcription factors in a target cell, which in turn aid in the amplification of the viral particles and viral replication [44,45].

EVs can also be used by viruses to deliver proteins into a given cell to increase the susceptibility of cells to viral infection. For example, HIV proteins (Nef-HIV) have been found in EVs and EV-mediated delivery of the proteins to recipient cells has been shown to induce the spreading of HIV-1 infection, making the cells more susceptible to HIV-1 infection [46,47]. In addition, researchers also found that Nef-HIV packaged in EVs released by macrophages could make cells less susceptible to cytotoxic immune response by degradation of lysosomal compartments in CD4+ T cells [48]. Nef protein thus enhances HIV-1-induced antibody abnormalities by attenuating expression of CD40L on CD4+ T cells, by hindering CD4+ T cell interactions with antigen-presenting cells, and by augmenting nonspecific B cell activation via macrophages. In line with these findings, according to Xu et al. [49], EVs containing Nef-HIV have a profound influence on T cells (adaptive immune response), via their ability to mediate evasion of adaptive immunity.

Another mechanism by which EVs increase the susceptibility of cells to viral infection is by shuttling viral receptors to recipient cells, thus facilitating viral binding to the cell and thereby increasing virus infectivity. This mechanism has been investigated in in vitro studies wherein EV-mediated delivery of HIV receptors has been shown to facilitate the entry of HIV-1 in target cells [7]. This mechanism of viral entry, however, has not yet been reported in vivo, and hence is a topic of future investigations. In some studies on Cytomegalovirus (CMV) and Herpes Simplex virus 1 (HSV-1) infections, researchers have demonstrated that EVs aid in increasing viral infectivity by the elimination of host proteins that are critical for antiviral defense. For example, CMV infection increases the release within EVs of proteins such as lectin and dendritic cell-specific intercellular adhesion molecule-3 grabbing non-integrin (DC-SIGN), which are necessary for virus uptake [50]. In contrast, in the case of HSV-1 viral miRNAs, miR-H28 and miR-H29 were found to have accumulated in latent cells, which, in turn, assists reactivation of the virus. Interestingly these viral miRNAs were absent in cells harboring the latent virus [51]. Reciprocally, overexpression of these two miRNAs in cells prior to infection was shown to reduce the accumulation of viral proteins, decreased plaque size, and reduced viral yields in cells infected with low multiplicity of infection. Furthermore, in these studies, it was also demonstrated that miR-H28 and miR-H29 could be exported from infected to uninfected cells prior to or concurrently with the entry of the virus into newly infected cells via EVs, resulting in reduced synthesis of viral gene products and viral spread across cells [51]. These findings thus suggest that HSV-1 downregulates its replication to enhance its spread by removing viral miRNAs in EVs [51].

### 2.4. Difficulties in Virions and EV Separation

In order to separate the functions carried out by the EVs and virions produced from the same cells, it is of paramount importance to separate the two populations. During earlier times, the separation of EVs and virions was found to be rather difficult, firstly because EVs and some virions (virions of retroviruses) are of similar sizes, with the EV size being in the range of 50–100 nm and virions being ~100 nm [9]. Secondly, both EVs and virions (virions of retroviruses) have similar buoyant density in the range of 1.13–1.18 g/L (EVs 1.13-–1.18 g/L and most retroviruses 1.16–1.18 g/L), hence making them inseparable using density gradient methods of separation. In order to advance EV biology in the context of characterization, recent advances have now allowed the separation of EVs from the virions using the velocity gradient approach. In this method, separation of particles is based on migration differences in a velocity gradient [52]. In addition, EVs can also be characterized and distinguished from virions by the presence of markers specific for EVs (Alix, TSG101, CD9, CD63 etc.) that are unlikely to be present in most viruses [22,23]. Furthermore, recent advances in flow cytometry-based separation of EVs that is based on particle characteristics and membrane antigen expression could also allow for better separation of EVs from the virions [53,54,55].

## 3. EVs in Viral Infections Affecting the Nervous System

**Figure 2:**
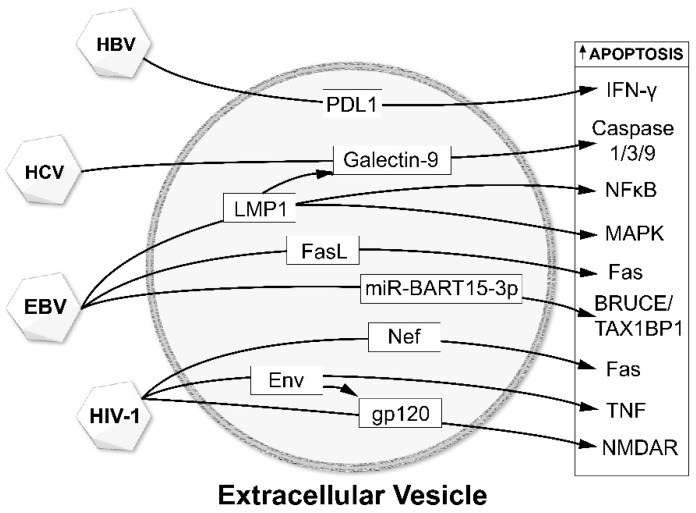
Pro-apoptotic effects of viruses on EV cargo. Several viruses including hepatitis B virus (HBV), hepatitis C virus (HCV), Epstein-Barr virus (EBV), and human immunodeficiency virus-1 (HIV-1) induce their pro-apoptotic effects through signaling molecules that are carried by EVs, which, in turn, act on a variety of apoptosis signaling pathways in the recipient cells.

As described above, EVs and viruses have been shown to interact with each other and the environment in several ways: (a) viral infections alter both EV release and the content of their cargo; (b) viruses use cellular EV machinery for production of progeny viruses; (c) EVs and their cargo play essential roles in propagating viral infection and transmission. The interactions between EVs and viruses affect disease progressions such as target cell apoptosis (Figure 2) and viral infection-mediated neurodegenerative diseases. The viruses chosen for this review are those that most commonly affect the central nervous system either directly (e.g., Zika virus) or indirectly (e.g., viral hepatitis-induced encephalopathy).

### 3.1. EVs in HIV-1 Infection

As per the United Nations Programme on HIV/AIDS 2017 data, 36.9 million people are currently living with HIV-1 infection globally. With the advent of combined antiretroviral therapy (ART), while the mortality rate of HIV infected patients has reduced dramatically, there is a concomitant increase in the lifespan of these infected individuals [56]. HIV virus has been reported to hijack EV biogenesis and its release from the infected cells. Both the HIV virions and HIV-infected macrophage-derived EVs are similar in size and both carry host proteins, indicating their origins from a common source [57]. Several reports have shown that EVs can also carry HIV viral proteins such as Tat, Nef, and gp120 [58,59,60]. HIV has also been shown to regulate the packaging of mRNAs, proteins, and cytokines in EVs [61,62]. Recent reports have also demonstrated a change in the proteomics profiling of plasma EVs derived from patient samples. Results have shown that fibulin-1, was significantly altered in HIV+ smokers, while hemopexin was altered in only HIV + drinkers. This study also showed that properdin expression was downregulated in the plasma exosomes isolated from HIV + smokers and drinkers compared to those from HIV-negative patients. This study thus demonstrated that hemopexin and properdin could be considered as physiological markers in alcohol and tobacco abusing HIV-infected individuals [63]. Another interesting study has shown that CSF EVs derived from neurons, glial cells and choroid plexus of HIV+ individuals with cognitive impairment carry synaptic, inflammation-related and stress response proteins, thereby underpinning their role as potential biomarkers for HIV-associated neurocognitive disorders (HAND) [64].

It has been reported that EVs could enhance HIV-1 infection by mediating the cellular transfer of co-receptors (CCR5) involved in viral entry, specifically via transfer to CCR5-deficient PBMCs and endothelial cells [65]. Similarly, platelet- and megakaryocyte-derived EVs were also shown to shuttle CXCR4 receptors to CXCR4-null cells [66]. Other studies have shown that binding of the EV PdtSer receptor TIM-4 to the surface of HIV-1 could also potentiate EV entry leading, in turn, to HIV-1 infection [67,68]. This is supported by the finding that the blockage of TIM-4 with specific antibodies inhibited EV-induced HIV entry in human T-cells and macrophage cell lines [68]. Interestingly, PtdSer moieties were also found on the EVs and/or HIV-1 particles in the serum of infected individuals, thereby implying potential systemic pro-viral effects beyond cell entry (reviewed by [69]. All the EV-mediated effects on host cell surface proteins could thus favor viral dissemination, leading to the infection of cells, even in cells which inherently do not express endogenous HIV-1 co-receptors. 

In addition to affecting viral entry through cell surface proteins, EVs can also facilitate HIV-1 infection by several other mechanisms. For example, EVs can act as carriers for the HIV virions, thus shielding the virions from immune surveillance. Interestingly, in the supernatant fluids of infected macrophages, HIV-1 was found to be in close physical association with EVs when assessed by electron microscopy. EVs isolated from infected cell supernatant fluids was able to potentiate infection to a greater extent than by virus itself. Thus, EVs have the potential to facilitate viral infections via several cell surface receptors and adhesion proteins [70]. EVs also play important roles in transferring bioactive molecules derived from HIV-1 such as HIV-1 envelope (Env) protein gp120, which is important for viral infectivity, into human lymphoid tissues [58]. HIV-1 Gag has also been found to be oligomerized with the EV membrane, thereby allowing for the facilitation of viral protein entry [71,72]. 

HIV-1 Nef protein, a major determinant of HIV-1 pathogenicity (reviewed by [73]) has also been found in the EV cargo as well as in the plasma of HIV patients, despite undetectable levels of HIV-1 RNA [47,74]. There are also reports indicating that viral protein Nef potentiates EV biogenesis [59]. EVs carrying Nef have been shown to induce apoptosis of CD4+ T cells, in turn leading to T cell depletion during infection [59]. Interestingly, EVs containing Nef, ADAM17 and pro-inflammatory mediators were shown to correlate with HIV-1-associated immune pathogenesis [75,76]. In vivo hepatocytes were considered to be the major source of Nef/ADAM17 pro-inflammatory EVs [75]. 

In addition to proteins, HIV infection can also induce expression of miRNAs in cells and in the packaging of EVs [77]. EVs derived from HIV-1-infected macrophages as well as plasma of HIV-1 infected patients were shown to exhibit increased levels of miR-88 and miR-99 in their cargoes. These EVs were also shown to stimulate TLR8 signaling, resulting in an increased release of TNFα, leading to chronic immune activation in patients [78]. Additionally, the pre-miRNA HIV-1 trans-activation response (TAR) was also found in the EVs isolated from supernatant fluids of HIV-1-infected cells and plasma from infected patients. These TAR-containing EVs could mediate infection in uninfected cells by reducing the expression of Bim and Cdk9 proteins in target cells [79]. A recent study demonstrated that although ART is able to reduce viral load to undetectable levels, it was ineffective in reducing the number of EVs containing viral products, which, in part, could explain the associated neurocognitive and immunological dysfunction observed in patients with HIV-associated neurocognitive disorders [80]. 

HIV-1, its viral proteins, as well as HIV-infected immune cells, are all capable of crossing the blood–brain barrier (BBB) leading to virus infection in the CNS (reviewed by [81]. There are reports suggesting the role of EVs in HIV-related neuroimmune pathogenesis. EVs from HIV-1-infected microglia are known to carry the viral Nef protein, which, in turn, can increase BBB permeability by reducing the expression of tight junction protein ZO-1 [82]. Additionally, these Nef-carrying EVs were also shown to induce TLR-4-mediated expression of cytokines and chemokines in microglia [82]. Plasma-derived EVs were also shown to deliver Nef mRNA and induce the expression of Nef in a neuroblastoma cell line, resulting in the increased production and secretion of neurotoxic Aβ peptides, which, in turn, could contribute to HAND [82].

EVs can also be used by the host to protect against HIV-1 infection. For example, EVs isolated from breast milk [83] and semen [84] have been shown to exhibit antiviral activity during HIV infection. There are also reports demonstrating that culturing HIV producer cells in EV-depleted media limits HIV-1 production [85]. Interesting studies have also shown that rapamycin, an inducer of autophagy, inhibits HIV-1 replication [86], likely by limiting EV biogenesis. A major drawback of rapamycin therapy, however, is its immunosuppressive effects, which limit its potential use for the treatment of HIV-1. Synthetic EVs, however, owing to their low immunogenicity compared to liposomes or lentiviral-based delivery systems, can be envisioned as promising tools for the delivery of RNA therapeutics aimed at blocking HIV infection. In recent years, many clinical trials aimed at assessing the therapeutic potential of EVs in various diseases, including Parkinson’s disease and cancer, have been extant; however, no consistent efficacy has been conclusively demonstrated to date (reviewed by [87]).

### 3.2. EVs in HTLV Infection

Human T-cell leukemia virus-1 (HTLV-1) is a blood-borne retrovirus estimated to infect ~5 to 10 million people worldwide. HTLV-1 is primarily transmitted by cell-to-cell communication. The EVs released from HTLV-1 infected cells can carry cargoes containing viral proteins (Tax, HBZ, gp61) and RNA, which, in turn, can alter the functions of the uninfected recipient cells by impairment of autophagy or other vital cellular functions [88]. In this study, it was also demonstrated that HTLV-1 derived EVs on their own were not infectious when tested in multiple recipient cell lines. In vivo studies, however, have shown that administering HTLV-1 EVs to NOG mice resulted in increased viral RNA synthesis in mice compared to the administration of EVs from uninfected cells [88]. Proviral DNA levels in these mice were increased in blood, lung, spleen, liver, and brain following treatment with HTLV-1 EVs, thus underpinning the role of HTLV-1 EVs in disseminating to different tissues, ultimately leading to its pathogenic effects [88]. In another study, it was shown that EVs derived from HTLV-1-infected cells contained cargoes such as Tax protein, proinflammatory mediators, and viral mRNA transcripts, including Tax, HBZ, and Env [89]. Furthermore, this study showed that exosomes released from HTLV-1-infected cells carrying Tax protein enhanced the survival of Fas antibody-treated cells [89]. Additionally, Tax-containing EVs protected IL-2-dependent CTLL-2 cells from apoptosis through activation of AKT [89]. Another study demonstrated the presence of the Tax protein in HTLV-1-EVs isolated from patient PBMCs and CSF, despite the undetectable level of virus in the CSF. These EVs were capable of sensitizing target cells for HTLV-1 specific CTL lysis [90]. Similarly, another study also showed that CSF-derived EVs from HTLV-1 patients carried the Tax protein despite undetectable viral levels (Detection of Human T-cell Lymphotropic Virus Type I proteins in exosomes from HAM/TSP patient CSF by novel nanotrap technology (I7-5E) Monique Anderson, Benjamin Lepene, Fatah Kashanchi, Steven Jacobson, Neurology Apr 2015, 84 (14 Supplement) I7-5E). Several studies have shown that HTLV-1 EVs carry viral proteins and proviral DNA, thereby underscoring further interest in the field of HTLV-derived EVs and their ability to impair functionality of the recipient cells.

### 3.3. EVs in Zika Infection

Zika virus (ZIKV) is a neurotrophic RNA flavivirus which is transmitted to humans primarily through the bites of infected *Aedes* mosquitoes via both vertical/perinatal and sexual transmission in humans. ZIKV infection during fetal development can result in brain abnormalities in the offspring. Reports have shown that mosquito EVs released from ZIKV-infected (C6/36) cells carry viral RNA and ZIKV-E protein cargoes and can infect naïve mosquito and mammalian cells. The ZIKV C6/36 EVs can lead to the differentiation of naïve monocytes and induce the expression of pro-inflammatory cytokines. Additionally, the ZIKV C6/36 EVs have also been shown to cause damage to vascular endothelial cells and induce inflammation, leading to increased BBB endothelial permeability [91]. Another study demonstrated that ZIKV could infect human astrocytes derived from fetal brains and that neurons were less susceptible to ZIKV infection compared with astrocytes. The infected astrocytes released viral particles and also significantly enhanced EV release. Interestingly, pre-treatment with GW4869, an inhibitor of neutral sphingomyelinase-2, decreased EV levels with concomitant suppression of ZIKV propagation, while also inhibiting infectious virions in astrocytes [92]. 

It has been reported that ZIKV uses exosomes as mediators for viral transmission between neurons. Electron microscopy analysis has shown neuronal exosomes of varied sizes, ranging 30–200 nm, following ZIKV infection. ZIKV infection enhanced the release of exosomes from neuronal cells, which not only carried ZIKV viral RNA and proteins but also showed time-dependent packaging of the viral contents in the EVs, which were also able to infect naïve cells. This study also showed that ZIKV induced the gene expression of neutral Sphingomyelinase (nSMase)-2/SMPD3, which regulates EV biogenesis and release. ZIKV modulates SMPD3-mediated EV biogenesis/release from cortical neurons and leads to transmission of viral contents through exosomes. It was also shown that these exosomes could increase infectivity and neuronal death, which, in turn, could underlie neurological manifestations such as microcephaly in the developing embryonic brains [93]. 

Studies have also demonstrated that ZIKV can be packaged in the cargo for the placental exosome pathway at the trophoblast endoplasmic reticulum, which is associated with the “secretory autophagy” process [94,95]. Distinct from the degradative autophagy (fusion with the lysosome), the non-degradative autophagic machinery, also known as the secretory autophagy, could result in the secretion of virus particles rather than their degradation [96]. Infection with ZIKV was shown to result in secretory autophagy leading to the release of vesicle cargoes containing additional virus particles [97], suggesting that regulation between degradative and secretary autophagy is essential for ZIKV transmission and infectivity [98]. Taken together, it is evident that ZIKV can be transmitted through EVs in the brain and that these EVs containing ZIKV could, in turn, influence the BBB permeability as well as periphery, thereby opening up future avenues for research in this field.

### 3.4. EVs in CMV Infection

Human cytomegalovirus (CMV), is a member of the Herpesvirus family [99] which encodes several viral envelope proteins [100], including gB and gH, which together with a third glycoprotein, gL, represent the fusion complex of this virus and are essential for viral entry [101]. A recent study has shown that CMV-infected cells release EVs, of which 15% carried gB cargoes, while 5.3% were positive for gH and 3.74% were positive for both gB and gH [102]. Additionally, other studies have also shown that exosomes derived from CMV-infected human endothelial cells can activate memory CD4+ T cells isolated from CMV-infected donors, via the transfer of antigen to allogenic DCs [103]. In infections with CMV, EVs released from the infected cells contain soluble DC-SIGN, a C-type lectin family molecule that increases the susceptibility of recipient cells to CMV [50]. While some reports have shown that the ESCRT machinery is essential for incorporating the viral cargo into MVBs [104], others have suggested that CMV can undergo maturation independent of ESCRT components [105]. Studies by Cepeda et al., have demonstrated that CMV infected cells release vesicles that traffic between the trans-Golgi network (TGN) and endosomes, in turn, carrying the TGN and endosomal markers (TGN46, annexin I, CD63, endosomal marker early endosome antigen 1, transferrin receptor, and the cation-independent mannose 6-phosphate receptor) [106]. Although studies have shown that CMV can induce EV biogenesis/release, which, in turn, can increase the susceptibility of infection in the recipient cells, more studies are warranted to better understand the role of EVs in CMV infection and its pathogenic effects.

### 3.5. EVs in Epstein-Barr Virus Infection

Epstein–Barr virus (EBV, also known as human herpesvirus 4) is a large enveloped oncogenic herpesvirus that plays a key role in the pathogenesis of multiple malignancies, including Burkitt’s and Hodgkin’s lymphomas, nasopharyngeal carcinoma and gastric carcinoma. The virus primarily infects B-lymphocytes and establishes long-term latency.

Two of the viral oncoproteins, latent membrane protein 1 (LMP1) and LMP2A, can be incorporated into the exosomes secreted from the infected host cells. LMP1 is likely related to immunosuppression [107,108,109] and exists primarily in the Golgi apparatus intracellularly and on the exosomes extracellularly [110]. Increased levels of LMP1 in exosomes are associated with higher levels of exosomal galectin 9 [109]. Although LMP1 alone appears to have a larger effect on T cell proliferation compared to galectin 9 [109], LMP1-negative exosomes expressing galectin 9 are still able to significantly increase apoptosis in EBV-reactive cytotoxic CD4+ T cells [111]; it is thus likely that LMP1 and galectin 9 act in a coordinated manner.

Major changes have been seen in the B cell exosomal proteome following infection by EBV compared with uninfected parental cell lines [112]. In this study, 93 of the differentially expressed proteins were uniquely expressed in EBV exosomes compared to the uninfected cells or a comparison group infected by Kaposi sarcoma herpes virus. The differentially expressed proteins included both the α and β chains of the HLA-DR receptor, suggesting that EBV could affect cell surface markers. Other unique differentially expressed proteins were mostly related to interferon and NF-κB signaling, membrane and protein trafficking, lipid raft organization, and cellular/vesicle binding. Exosomes isolated from EBV-transformed B-cells also contain Fas ligand (FasL) and MHC class II molecules and can induce cell death of T-helper cells [113]. This death occurs in both a dose- and time-dependent manner. Exosomes released by the non-infected cells of the same cell line failed to generate similar apoptotic effects [114].

In addition to proteins, the exosomes secreted by EBV infected B-cells also contain high quantities of RNA. One study assessing miRNA expression demonstrated between 10^2^ and 10^5^ copies of EBV miRNAs per ng of exosomal RNA. The EBV viral miRNAs were selectively enriched in exosomes and their concentration in exosomes was found to be almost four-fold higher compared to the host cell intracellular levels [107]. EBV-related exosomal small RNAs included the miRNAs EBV-miR-BART3 and EBV-miR-BHRF1-1 as well as the lncRNAs H19 and H19 antisense [115]. The exosomal EBV miRNAs have been shown to successfully transfer into uninfected recipient cells, affecting the target genes within the recipient cells [107]. These miRNAs can regulate the immune response by downregulating CXCL11/ITAC in the target cells. These EBV miRNAs have also been found to be present in significant quantities in non-B-cells in ~60% of asymptomatic HIV-EBV co-infected patients with elevated EBV viral loads [116].

EBV-encoded small RNAs (EBERs) target toll-like receptor 3 and can bind to a protein, lupus antigen (La), which is found in purified exosomal fractions. EBER1 and EBER2 have been shown to be released in exosomes from EBV-infected cells [117]. Cytoplasmic EBER1 is selectively sorted into exosomes that are internalized by host cells, including dendritic cells, and can trigger antiviral immunity [118].

The specific role of EVs released by EBV-infected cells in the transmission of the virus or the pathogenesis of brain injury remains unclear. However, given the known effects of the EBV-induced EV cargo, several hypotheses have been proposed. For example, both LMP-1 and EBERs have been associated with neuroinflammation and injury, based on the fact that their expression can be detected in more than 85% of brains with multiple sclerosis lesions but rarely in healthy control brains [119]. Additionally, upregulated levels of miR-146a in EBV infection [120] predicted the clinical course (hazard of conversion and relapse, as well as annualized change in disability) of patients with multiple sclerosis [121].

### 3.6. EVs in Hepatitis B and C Virus Infection

Although not a result of direct viral infection of the CNS, infection with hepatitis B (HBV) or C virus (HCV) can also present a neurological phenotype due to the development of hepatic encephalopathy. Hepatitis B virus (HBV) is a partially double-stranded enveloped DNA hepadnavirus which exclusively infects hepatocytes. Three primary types of subviral particles are produced in HBV-infected patients. The first type of particles contains complete HBV virions [122], with the other two consisting of two types of EVs carrying incomplete HBV virions. The first EV type is composed of host lipids and the viral surface antigens only without the viral capsid or nucleic acids [123], and the other is an “empty virion” which consists of the viral envelope and capsid proteins but no nucleic acids [124]. HBV-encoded exosomes contain HBV DNA [125], RNA and proteins [126] and have been associated with several different cellular effects. EVs secreted by HBV-infected hepatocytes can target peripheral blood monocytes, upregulating programmed death ligand-1 (PD-L1), resulting in a suppressed T cell response through downregulation of CD69 [127]. Within the monocytes, EVs released from HBV-infected hepatocytes could induce expression of the NKG2D ligand by stimulating MyD88, TICAM-1, and MAVS-dependent pathways [126]. A significant component of the effects of HBV on human immune response relies on EV-mediated viral transmission into NK cells resulting in NK cell dysfunction, in part, due to the suppression of the nuclear factor-κB (NF-κB) and p38 mitogen-activated protein kinase (MAPK) signaling pathways [128]. HBV infection also results in an increase in EV and exosomal immunoregulatory microRNA levels. These miRNAs are transferred to macrophages, resulting in suppression of IL-12 expression to counteract the host innate immune response [126].

Hepatitis C virus (HCV) is an enveloped non-arbovirus member of the *Flaviviridae* family that is primarily transmitted by blood-to-blood contact or sexual intercourse. HCV virions are very small particles (~50 nm in diameter) and have been isolated from EVs secreted by HCV-infected human hepatoma cell lines [129,130]. HCV transmission can occur both in association with exosomes or via an exosome-independent pathway [131]. Despite the fact that HCV delivery within exosomes can protect the virus from host recognition, EVs containing HCV RNA still result in an innate IFN-alpha response in neighboring dendritic cells (DCs) [132]. Additionally, both exosome-free HCV and exosomes containing HCV increase the expression of TLR7/8 in circulating monocytes [133]. Despite both of these host-protective mechanisms remaining intact in HCV infection, circulating EVs that contain HCV virions continue to promote HCV transfer, as suggested by the spread of HCV despite the presence of neutralizing antibodies [129,134].

In addition to HCV virions, EVs secreted from HCV-infected cells can also carry other cargo which, in turn, relates to the deleterious effects of the virus. For instance, EVs secreted by HCV-infected hepatocytes carry miR-19a which targets SOCS3 in hepatic stellate cells (HSCs), resulting in enhanced expression of the fibrosis marker genes through the STAT3-mediated transforming growth factor β signaling pathway [135]. HCV infection also induces the expression of miR-192 within EVs that are similarly taken up by HSCs. The up- or down-regulation of miR-192 results in respective increase or decrease in fibrogenic markers, in targeted HSCs [136]. CD81 has also been shown to be elevated in exosomal serum fractions of patients with chronic hepatitis C, and the CD81 levels correlated with ALT levels and severity of liver fibrosis [137]. Furthermore, the presence of a miR-122, HSP90, and Ago2 complex in exosomes has been shown to increase receptor-independent HCV transmission to naïve cells [138].

### 3.7. EVs in JCV Infection

The John Cunningham (JC) polyomavirus (John Cunningham virus, JCV, JCPyV) is a small non-enveloped double-stranded DNA virus that is present in around half of the population of the world. It can spread to the CNS of immunosuppressed patients, leading to oligodendrocyte demyelination resulting in progressive multifocal leukoencephalopathy (PML). JCV infects cells of the choroid plexus through a two-step process involving binding to both the sialic acid moiety of lactoseries tetrasaccharide C (LSTc) and a serotonin receptor. Oligodendrocytes and astrocytes, however, which are also common targets of JCV CNS infections, do not express LSTc and do not bind the virus, thus suggesting an additional receptor-independent mechanism of infection. 

Several viruses (mostly RNA viruses) have been shown to use EVs as infectious delivery systems, though non-enveloped DNA viruses have not commonly been thought to utilize EVs until recent studies demonstrated that JC virions were present inside, and occasionally on the surface of, EVs secreted from JCV-infected epithelial cells in the choroid plexus [139,140]. JCV virions are around 40 nm in diameter [141], and thus are easily capable of inclusion within EVs that are up to 1000 nm in size. EV-mediated infection is not neutralized by antiviral antisera and appears to be independent of the presence of the LSTc attachment receptor or serotonin entry receptor [139]. The EVs are taken up by recipient cells through both clathrin-dependent endocytosis and micropinocytosis [140]. 

The JCV-encoded miRNAs mir-J1-5p and miR-J1a-5p have been shown to be useful biomarkers for JCV infection and can be detected in the stool [142], serum and urine [143] even in VP1 serology negative patients. Additionally, JCV-miR-3p downregulates ULBP3 expression in order to inhibit clearance of the infected cells by NK cells [144]. It is likely that these JCV-related miRNAs are secreted within EVs, as the majority of detectable miRNAs in serum are concentrated in EVs [145]. The presence of these JCV-encoded miRNAs within EVs, however, has not yet been directly investigated and warrants further research. 

Although miRNAs are a key component of EVs and similar miRNAs have been shown to be excreted in polyomaviruses such as the BK virus [146], it cannot be assumed that the JCV miRNAs are similarly transported since other JCV products that could undergo EV transport do not appear to do so. A good example of this is the regulatory phosphoprotein agnoprotein, which is important for the successful propagation of JCV and plays a vital role in viral DNA replication. Agnoprotein is absent in EVs secreted by JCV-infected cells yet is found in high concentrations in EV-depleted serum [147].

### 3.8. EVs in Herpes Simplex Virus Type-1 Infection

Herpes simplex virus type-1 (HSV-1) is one of the most prevalent human pathogens capable of infecting both epithelial cells as well as neurons, ultimately establishing latent infection in the trigeminal ganglia [148,149]. It is a DNA virus belonging to the Alphaherpesvirinae subfamily within the Herpesviridae family which is known to have a highly complex genome encoding several enzymes needed for its biphasic life cycle. HSV-1 is known to cause both oral/labial and genital lesions [150,151,152,153,154]. HSV-1 has also been reported to cause encephalitis or keratoconjunctivitis [155,156,157].

Dating back to the early years of HSV discovery, HSV infected cells have been shown to secrete small vesicles that were originally called L-particles. These L-particles are noninfectious and lack the viral capsid and DNA [158,159,160]. The biogenesis of these L-particles involves the MVB pathways and, similar to exosomes, have also been shown to deliver functional cargo to the bystander cells [161,162]. In one study, it was shown that transfected HSV viral DNA could form infectious foci or plaques in the presence of L-particles, thereby suggesting enhanced replication [161]. One of the HSV-1 viral proteins found in the L-particles is the tegument protein that functions to prime naïve cells for infection; by inference, then, L-particles could function as enhancers of viral infectivity/replication [162]. Additionally, Heilingloh et al., demonstrated that L-particles could transmit viral proteins from HSV-1-infected mature dendritic cells to uninfected cells, resulting in decreased expression of CD83 that is known to have costimulatory properties, in turn, suggesting a role of L particles in escape of the immune response and increased viral infectivity [163]. 

The highly complex HSV-1 genome encodes several mature miRNA sequences [164]. These HSV-1 derived miRNAs are thought to be involved in repressing the expression of target genes critical for the regulation of viral latency [164,165]. Interestingly, EVs from HSV-1 infected cells have also been shown to contain both viral mRNA and miRNA such as miR-H28 and miR-H29 [51,166]. While these miRNAs are ectopically expressed in human cells before infection, they reduce the accumulation of viral mRNAs and proteins. In addition, they are more abundant in cells where latent reactivation occurs. This evidence thus indicates that HSV-1 utilizes strategies to regulate its own expression to maximize its spread [51]. Additionally, these studies also showed that HSV-1 infected cells released EVs that carried the human host antiviral factor Stimulator of INF genes (STING), which contributes to restricting viral spread and, in turn, promotes host cell survival [166,167].

HSV has been shown to evade the immune system, in part, by modulating the major histocompatibility complex (MHC) class II antigen-processing machinery by shuttling HLA-DR to the exosomal secretion pathway instead of to the cell surface [168,169,170]. Another mechanism of evading the immune system was demonstrated when EVs containing HSV-1 virions were not neutralized by anti-HSV-1 antibodies, thus suggesting that EVs shielded the virus from neutralization [171].

HSV-1 is known to infect epithelial cells and subsequently travel to neurons and establish latent infection in the trigeminal ganglia. Another study has demonstrated infection of human oligodendroglial (HOG) cell line by HSV-1 [171]. Neural cell types are known to release EVs, which play important roles in regulating synaptic activity and cell-to-cell communication [172,173]. Using transmission electron microscopy (TEM), Bello-Morales et al., showed that HSV-1 virions were transferred from infected to uninfected oligodendrocyte cells via microvesicles, thus suggesting that these microvesicles could be involved in both viral spread and likely could also contribute to the evasion of immune surveillance [171].

As discussed herein, HSV-1 can infect both peripheral and CNS cells leading to the production of L-particles from infected cells carrying host and viral proteins and miRNAs. Research suggests that these L-particles and EVs mediate cross-talk between the periphery and CNS to facilitate infection and immune evasion. Detailed mechanisms, however, remain to be unraveled.

The roles of EVs in different viral infections are summarized in Table 1. 

## 4. EVs as Biomarkers and Therapeutic Vehicles for Viral Infectious Diseases

EVs and their cargo in circulating systems serve as ideal biomarkers for various neurodegenerative and infectious diseases, owing to the ability of EVs to cross biological barriers, such as the BBB and the gut barrier. As such, tissue- and organ-specific EVs are able to reach the peripheral circulation in adequate concentrations to allow for clinically relevant measures to be performed. This property of EVs also makes them promising delivery vehicles for carrying therapeutic molecules for the treatment of various diseases.

### 4.1. EVs as Biomarkers for Viral Infectious Diseases

The expression of and change in pathogen-derived factors in the EVs can serve as diagnostic biomarkers and indicators of disease progression. The change in EV cargoes during disease progression makes them excellent biomarker candidates. In January 2016, the first commercially available blood-based EV test for cancer diagnosis in the US was marked as a major step in the maturation of EVs as diagnostic factors [181]. Research into the development of EV-derived diagnostic biomarkers for infectious disease is relatively new, but shows immense promise, for several reasons. First, the diagnosis of intracellular viral pathogens normally requires the culture of pathogen samples derived from infected tissue, followed by its molecular analyses. This method is dependent on the type of sample collection and the time of collection prior to testing, which can result in misdiagnoses, especially in patients with a low pathogen load [182]. EVs containing pathogen-derived factors are actively released from most cells and, in turn, accumulate in the circulation. Some studies indicate that infection also increases EV release rates [137,183,184]. For example, the EV CD81 fraction in serum was found to be highly increased in patients with chronic hepatitis C, which was found to be associated with inflammatory activity and severity of fibrosis [137]. 

Emerging evidence indicates the potential of EVs isolated from non-invasive biological samples for detection of viral infections. HIV-1 infection is usually detected by antibodies to HIV—a method that can take months to develop—or by measuring viral loads in blood. As an alternative to traditional detection methods, researchers have developed newer assays to detect HIV-1 proteins in urinary EVs to provide a noninvasive method that achieves rapid screen for infection [185]. In addition, increased numbers of CSF EVs have also been detected in HIV-positive individuals. An increase in concentrations of CSF EVs has been shown to correlate with an established neuronal injury biomarker (neurofilament light chain protein (NFL) as well as progression to HAND [186]. Furthermore, our group isolated neuronal-derived EVs (L1CAM+ EV) and found an increased presence of L1CAM+ neuronal-derived EVs both in the brain and serum of HIV-1 transgenic (Tg) rats, a model that results in the expression of HIV viral proteins but without active viral replication [187]. In another study the authors performed mass spectrometry on neuronal-derived EVs from HIV-infected subjects and detected a number of proteins that were associated with synapse organization, synaptic signaling, cognition, and neurogenesis such as APP, L1CAM, NCAM, neprilysin, and NF-L, thus implicating that neuronal-derived EVs could serve as potential biomarkers for HAND, Alzheimer’s disease and other neurodegenerative disorders [188]. Understanding the source and profiling cargoes (miRNAs or proteins) of CNS-derived EVs, and their relationship with neurocognitive impairment in HIV-positive individuals with cART could further contribute to the discovery of novel biomarkers.

### 4.2. EVs as a Therapeutic Vehicle for Viral Infectious Diseases

Many features of EVs allow them to regulate the pathogenesis of infectious pathogens as well as allows them to serve as effective agents that could be used for the development of novel therapeutic approaches [14]. Owing to the characteristics of EVs such as stability in circulation, biocompatibility, as well as low immunogenicity and toxicity, these vesicles have become attractive systems for the delivery of therapeutics. Additionally, the ability of EVs to selectively deliver molecules to specific recipient cell types achieves a number of advantages using EVs as the basis for a pathogen-specific therapeutic approach [189,190,191,192,193]. Various pathogen-derived factors can be carried by EVs, such as receptors involved in cell targeting or recognition, suggesting the possibility of modifying EVs to achieve targeting of specific cells of interest. For example, Alvarez-Erviti et al. fused the neuron-specific rabies viral glycoprotein (RVG) with Lamp-2b to target EVs to neurons. These targeted EVs could cross the BBB and deliver functional cargo (exogenously loaded siRNA), resulting in gene knockdown selectively in neurons [16].

Currently, liposomes have been widely used as a drug delivery system as carriers of therapeutics to target tissues and cells. However, this method has several drawbacks, including poor in vivo stability and retention, the challenge of drug loading, leakage and release, and the low selectivity of therapeutics-loaded liposomes to specific tissues and cells [194]. Elegant studies have demonstrated that during EV biogenesis, diverse factors can be selectively packaged, which, in turn, results in selectively targeting multiple cells and tissue types via the interaction of specific membrane factors [16,195,196]. Many in vitro studies have shown that EVs can be used for the delivery of exogenous nucleic acids, such as miRNA and anti-miRNA to target cells [197,198]. For example, isolated EVs from human primary astrocytes can be loaded efficiently and can transfer miR-7 to neuron, leading, in turn, to the induction of synaptic injury [197]. Increasing evidence has shown that EVs are promising conduits for delivery of RNA therapeutics (reviewed in [199]). For example, Cooper et al. have elegantly demonstrated that tail vein injection of modified exosomes (RVG-exosome) loaded with α-Syn siRNA could deliver siRNAs to the brain and also lead to the reversal of α-Syn associated pathological manifestations in human S129D α-Syn transgenic mice [200]. Our group has also developed EV-loaded RNA drug target(s) as therapeutics. For example, our in vivo study, for the first time, demonstrated that intranasal delivery of lincRNA-Cox2 siRNA restored microglial phagocytic activity of morphine-administered mice [201] as well as LPS-induced microglial proliferation in mice [202]. These findings could have ramifications for the development of intranasal delivery of EV-loaded small RNA that could serve as therapeutics for a multitude of neurodegenerative disorders, including those associated with neuroinflammation.

EVs also can be efficiently taken up by antigen-presenting cells (APCs), indicating that these EVs can be useful for the delivery of antigens and co-stimulatory factors, that directly promote robust immune responses against a pathogen. For example, Cheng Y et al. demonstrated that mice injected with EVs (isolated from macrophages pulsed with M. tuberculosis culture filtrate proteins) produced antigen-specific CD4+ and CD8+ T cell activation responses similar to those in mice immunized with M. bovis BCG (the only accepted vaccination for Mtb), indicating that non-infectious EVs containing pathogen-derived antigens could potentially serve as an alternative method for pathogen-free vaccine approaches [203]. One of the physiologic limitations that will need to be overcome in the development of EV-based vaccines, however, will be the concern that many EVs could exert immunosuppressive effects [204].

## 5. Conclusions and Perspectives

It has now been widely established that EVs are not just the cellular trash receptacles that they were once thought to be, but rather that they play a vital part in the intercellular transfer of functional proteins, lipids, and genetic material. During both acute and chronic viral infections, many viruses take advantage of this signaling system, using EVs to carry molecules needed for viral attachment, amplification, and replication to the target cells. Additionally, virus infections could also result in alterations in the content of endogenous EVs as a means to decrease the host immune responses and/or change the microenvironment to one that is more hospitable to the virus. Due to their ability to pass through the BBB, EVs also represent one avenue for viral signaling to pass from the systemic circulation into the brain environment.

Virus-altered EVs are not just a mechanism for viruses to improve their ability to successfully replicate. They are also an opportunity for investigators to gain a window into the workings of the viral machinery. The altered EVs that can be found in the serum, urine, CSF, and stool are increasingly being used as biomarkers to identify the presence and severity of a viral infection. Additionally, EVs offer a biological, modifiable, targetable, and relatively stable vehicle for therapeutic molecules. This is a rapidly evolving field that holds great promise for future diagnostic and therapeutic interventions, potentially decreasing the rate or severity of virus-induced systemic and brain injury.

## Figures and Tables

**Table 1:** Extracellular vesicles in viral infectious diseases.

Virus	Model	EV Origin Cells	EV Cargo	EV Recipient Cells	EV Function	Ref.
HIV-1	*In vitro*	Macrophage	Host proteins			[57]
	*In vitro*	CCR5+ Chinese hamster ovary cells, PBMC	CCR5	PBMC, Endothelial cells		[65]
	*In vitro*	HIV virions	Env	Human Lymphoid tissues		[58]
	*In vitro*	8E5, ACH-2, U1, HLM-1, J1.1 cells and PHA and IL-2 activated peripheral blood lymphocytes	Dicer, Drosha protein, TAR RNA	J1.1 cells	Infection of the recipient cells	[79]
	*Ex vivo*	Patient serum	PtdSer			[69]
	*Ex vivo*	Hepatocytes	Nef/ ADAM17			[75]
	*Ex vivo*	Patient plasma	TAR RNA			[79]
	*Ex vivo*	Patient plasma	Nef mRNA	Neuroblastoma cell line	Expression of Nef and production of Aβ peptides	[82]
HTLV	*In vitro*	Human T-cell leukemia virus-1-infected cell lines HUT102, MT-2, and MT-4	Tax, HBZ, Env, gp61	uninfected T-cell (CEM and Jurkat), and promonocytic (U937) cell lines	Impairment of autophagy	[88]
	*Ex vivo*	Patient PBMCs and CSF	Tax protein		CTL lysis, Inflammation	[90]
	*Ex vivo*	Patient CSF	Tax protein			[90]
Zika	*In vitro*	C6/36 cells	Viral RNA and protein cargoes	Vacular Endothelial cells	Infect naïve mosquito and mammalian cells, endothelial cell permeability	[91]
	*In vitro*	Astrocytes	Virions			[92]
	*In vitro*	Neurons	Viral RNA and proteins		Neuronal death	[93]
CMV	*In vitro*	UL32-EGFP-HCMV-infected human lung fibroblast (MRC-5 cells), or AD169 HCMV-infected primary dermal fibroblast cells	gb and gH viral protein			[102]
	*In vitro*	CMV-infected human endothelial cells	Antigen	DC	Activate memory CD4+ T cells	[103]
	*In vitro*	Invitro generated DC culture	DC-SIGN			[50]
	*In vitro*	BJ1 cells	TGN46, annexin I, CD63, endosomal marker early endosome antigen 1, transferrin receptor, and the cation-independent mannose 6-phosphate receptor			[106]
EBV	*In vitro*	NPC cells	LMP1	T cells, Th1 lymphocytes, B cells	Activated PI3K/AKT and MAPK/ERK pathways and inhibits the function of immune cell	[109,111,174,175]
	*In vitro*	NPC cells	Galectin-9	T cells, Th1 lymphocytes	Induced apoptosis through interaction with Tim1 membrane receptor	[109,111]
	*In vitro*	Human B Cell-Derived Lymphoblastoid Cells	FasL	CD4+ T cells, B cells, epithelial cells	Induced cell death of T-helper cells	[113,114]
	*In vitro*	EBV-infected B cells	BHRF1	Dendritic cells	Downregulated CXCL11/ ITAC	[116]
	*In vitro*	Latently infected B cells	EBER1	Dendritic cells	Triggered antiviral immunity and decrease apoptosis of infected cells	[118]
	*In vitro*	NPC cells	EBERs	Endothelial cells	Promoted angiogenesis through VCAM-1 expression	[176]
	*In vitro*	NPC cells	HIF1alpha	NPC cells	Tumorigenesis	[174]
	*In vitro*	Gastric cancer cells	miR-BART15-3p	AGS-EBV cells	Induced apoptosis	[177]
HBV	*In vitro*	Hepatocytes	Viral RNA	Macrophages	Induced NKG2D ligand expression	[126]
	*In vitro*	Hepatocytes	miR-21, miR-29a	Macrophages	Suppressed IL-12p35 mRNA expression to attenuate NK cell response	[126]
	*In vitro*	Hepatocytes	Not specified	Monocytes	Upregulating programmed death ligand-1 (PD-L1), resulting in a suppressed T cell response through downregulation of CD69	[127]
	Human	Serum	Not specified	NK cells	Suppressed NF-κB and p38 MAPK signaling pathways	[128]
	*In vitro*		HBV X protein	Hepatocellular carcinoma cells	Increased risk of HBV-related HCC at least in part through the upregulation of miR-21 and downregulation of PDCD4	[178]
	*In vitro*	Hepatoma cell line Huh-7	VCP		Increased risk of HBV-induced HCC (theorized)	[179]
HCV	*In vitro*		Viral RNA	Monocytes	Increased the expression of TLR7/8	[133]
	*In vitro*		Not specified	Human monocytes	Increased galectin-9	[180]
	*In vitro*	Hepatocytes	miR-19a	Hepatic stellate cells	Targeted SOCS3 which enhances fibrosis marker genes through the STAT3-mediated transforming growth factor beta signaling pathway	[135]
	*In vitro*	Hepatocytes	miR-192	Hepatic stellate cells	Increased fibrogenic markers	[136]
	Human	Serum	CD81		Increased ALT levels and severity of liver fibrosis	[137]
	Human	Serum	miR-122, HSP90, Ago2 complex	Hepatocytes	Increased viral replication	[138]
JCV	*In vitro*		miR-3p	NK cells	Downregulated ULBP3 expression in order to inhibit clearance of the infected cells by NK cells	[144]
HSV	*In vitro*	BHK C13 cells	Viral tegument and envelope proteins	-	Non infectious light participles (L-particles) that enhance viral infectivity	[160]
	*In vitro*	Infected mature dendritic cells	viral proteins (ICP0, ICP4, gB, and MCP)	Naïve mature Dendritic cells	L-particles deliver viral proteins to modulate immune functions of uninfected bystander cells.	[163]
	*In vitro*	BHK C13 cells	Viral tegument	BHK C13 cells	L-particles can deliver functional tegument proteins	[162]
	*In vitro*	BHK C21 cells	Viral tegument	BHK C21 cells	Enhanced transfected HSV viral DNA replication (plaque formation)	[161]
	*In vitro*	HEp-2 cells	STING (stimulator of IFN genes); viral mRNAs; microRNAs	Vero cells	Silencing viral genes in latently infected neurons	[51,166]
	*In vitro*	Fibroblasts	STING, along with the EV markers CD63 and CD9	macrophages	Activated innate immunity in recipient cells and suppressed viral gene expression and virus replication.	[167]
	*In vitro*	human melanoma cell line (Mel JuSo)	Viral glycoprotein B (gB) & HLA-DR, CD63	-	Viral immune evasion involving hijacking of HLA-DR and releasing it in exosomes	[170]
	*In vitro*	Human oligodendroglial HOG cell line	HSV-1 virions	Chinese hamster ovary (CHO) cell line	MVs released by infected cells contain virions, are endocytosed by naive cells, and lead to a productive infection and reduce antibody-mediated neutralization	[171]

PBMC: Peripheral blood mononuclear cells; CTLs:Cytotoxic T Lymphocytes; DC: Dendritic cells; NPC: nasopharyngeal carcinoma; VCP: valosin-containing protein; ALT: alanine aminotransferase; BHK cells: Baby Hamster Kidney fibroblasts; HEp-2 cells: Human epithelial type 2; MVs: microvesicles.
